# Rigid‐Flexible Coupling Realized by Synergistic Engineering of the Graphitic‐Amorphous Architecture for Durable and Fast Potassium Storage

**DOI:** 10.1002/advs.202410966

**Published:** 2024-11-22

**Authors:** Mingchi Jiang, Ning Sun, Bin Cao, Xuyang Jian, Razium Ali Soomro, Bin Xu

**Affiliations:** ^1^ State Key Laboratory of Organic‐Inorganic Composites Beijing Key Laboratory of Electrochemical Process and Technology for Materials Beijing University of Chemical Technology Beijing 100029 China; ^2^ College of Materials Science and Engineering Xi'an University of Science and Technology Xi'an 710054 China; ^3^ Shaanxi Key Laboratory of Chemical Reaction Engineering School of Chemistry and Chemical Engineering Yan'an University Yan'an 716000 China

**Keywords:** amorphous phases, carbon anodes, graphitic structures, K‐storage mechanisms, potassium‐ion batteries

## Abstract

Graphite anodes hold great potential for potassium‐ion batteries (PIBs), yet their practical application is hindered by poor cycle performance caused by substantial interlayer expansion. Herein, a partial graphitic carbon (PGC) is elaborately engineered via the catalytic effect of ferric citrate using pitch as a carbon precursor. Systematically varying the catalyst content enables an optimal PGC design integrating a highly graphitized phase providing abundant active sites for K‐ion intercalation, balanced with an amorphous carbon region that accommodates volume expansion and facilitates ion diffusion. The optimized PGC12 electrode exhibits a high reversible capacity of 281.9 mAh g^−1^, characterized by a prolonged low‐potential plateau region, and excellent cycle stability with a capacity retention of 94.8% after 300 cycles. It also realizes an impressive rate capability with a retained capacity of 222.2 mAh g^−1^ at 1 C. Moreover, the assembled K‐ion full‐cell delivers an exceptional energy density of 148.2 Wh kg^−1^. In‐situ XRD and DFT simulations further verify the distinct phase transition mechanisms and reaction dynamics across different carbon configurations. This work elucidates the impact of carbon configurations on K‐storage performance and proposes a structural model for efficient K‐ion storage, which is instrumental in the rational design and advancement of carbon anodes in PIBs.

## Graphical abstract

A partial graphitic carbon (PGC) configuration integrating a highly graphitized phase with an amorphous carbon region is proposed for efficient K‐ion storage. The highly graphitized phase provides abundant active sites for K‐ion intercalation, while the amorphous carbon region accommodates volume expansion and facilitates ion rapid diffusion, thus synergistically improving the electrochemical K‐storage performances.

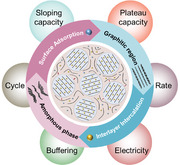

## 1 Introduction

Lithium‐ion batteries (LIBs) with the prominent advantages of high energy/power density and long cycle life have been extensively applied in various fields from portable electronics to electrical vehicles.^[^ ^1^, ^2^ ^]^ Nevertheless, the constrained availability and uneven distribution of lithium resources pose significant challenges to LIBs' future advancement and scalability in the development of large‐scale energy storage solutions.^[^ ^3^, ^4^ ^]^ As a result, developing alternative battery systems with abundant resources, environmental friendliness, and low cost has become increasingly indispensable.^[^ ^5^, ^6^, ^7^ ^]^ In this context, potassium‐ion batteries (PIBs) have garnered extensive attention owing to their high resource abundance (K with 1.5 wt% in earth crust vs Li with 0.0017 wt%), low cost, and relatively low redox potential of K^+^/K (−2.93 V vs SHE). These characteristics make PIBs a promising alternative to LIBs in grid‐level stationary energy storage systems (ESSs).^[^ ^8^, ^9^, ^10^ ^]^

Importantly, graphite, a commercial anode for LIBs, could also deliver a reasonable K‐storage capability with a theoretical capacity of 279 mAh g^−1^, which provides an opportunity to leverage existing LIBs technology to develop advanced PIBs.^[^ ^11^, ^12^ ^]^ However, the significantly large ionic radius of K^+^ (1.38 Å) compared to Li^+^ (0.68 Å) presents challenges when intercalating into the narrow interlayer spacing of graphite.^[^ ^13^ ^]^ This mismatch causes substantial structural changes in the graphite with a large volume expansion (≈60%) along the *c*‐axis during the intercalation process, leading to deteriorated structural stability and cycle performance.^[^ ^14^, ^15^ ^]^ In addition, the sluggish kinetics of K^+^ within the compact structure of graphite leads to a degraded rate capability of graphite anode, further restricting its practical usage in PIBs.^[^ ^16^, ^17^ ^]^

To date, enormous efforts have been made to improve the electrochemical performances of graphite and graphitic carbon anodes for PIBs, including reducing the particle size, constructing porous structures, heteroatoms doping, as well as enhancing the amorphous degree and enlarging the carbon interlayer spacing.^[^ ^13^, ^18^, ^19^ ^]^ However, most of these strategies tend to increase the capacities in the high‐potential sloping region at the expense of the low‐potential plateau region capacity and a considerable increase in irreversible capacity during the initial cycle, which significantly undermines the advantages and application potential of graphitic carbon materials in PIBs.^[^ ^20^, ^21^ ^]^ It is well recognized that the graphitization degree and the structural configurations of carbon anodes are critical in determining electrochemical potassium storage behaviors and performances. Pitch is a kind of attractive carbon precursor with high carbon content and low cost, and has been widely used to fabricate various carbon materials for different fields.^[^ ^22^, ^23^ ^]^ Typically, pitch‐derived carbon material exhibits a soft‐carbon nature, which allows full graphitization at temperatures above 2600 °C, and the carbon structure regulation could be easily achieved via the commonly used strategies at low temperatures.^[^ ^24^, ^25^, ^26^ ^]^ This capability makes pitch an ideal carbon precursor candidate for PIBs to further explore the correlation between the varied graphitic configurations and electrochemical K‐storage properties.

Herein, a range of pitch‐derived carbon materials featuring distinct graphitic‐amorphous architectures have been investigated to elucidate the correlation between the K‐storage performance and structural configuration, utilizing ferric citrate (FC) as a graphitization catalyst. A systematic study shows that compared with the pitch directly pyrolyzed carbon (MTP), the fabricated partial graphitic carbon (PGC) with the gradually increased graphitization degrees exhibited distinctively different K‐storage performances. Among, PGC14 with the highest graphitization degree delivered the highest initial reversible K‐storage capacity and initial columbic efficiency (ICE), but its excessive graphitization resulted in compromised capacity retention and rate performance. Conversely, PGC12 with a balanced highly graphitized carbon phase and the partly amorphous region demonstrated superior overall performances, achieving a reversible K‐storage capacity of 251.0 mAh g^−1^ after 300 cycles at 0.2 C, with an optimum rate capability of 179.5 mAh g^−1^ at a current density of 2 C. Additionally, the potassium‐ion full‐cell assembled using PGC12 as anode and PTCDA@450 as cathode delivers a high K‐storage capacity of 262.1 mAh g^−1^ and energy density of 148.2 Wh kg^−1^, reflecting the promising prospect of PGC anode for PIBs. Kinetic analysis coupled with density functional theory (DFT) simulation further confirms the critical role of partial graphitic carbon structure in efficient K ion storage, which might offer important guidance for the structural design and development of carbon anodes in PIBs.

## 2 Results and Discussion

**Figure 1:**
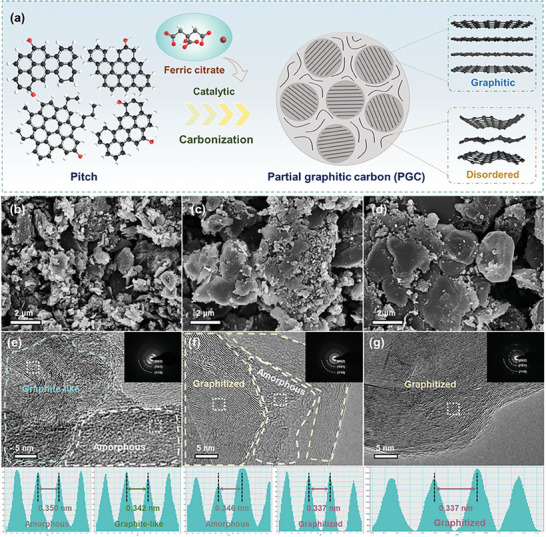
a) Schematic illustration depicting the construction of the partial graphitic carbon materials, SEM, HRTEM, SAED images with carbon interlayer spacing of b, e) PGC11, c,f) PGC12, and d,g) PGC14.

The fabrication process of the partially graphitic carbons (PGC) is schematically depicted in **Figure** **1**a. During the carbonization stage, ferric citrate (FC) decomposes into iron oxides and amorphous carbon at increasing pyrolysis temperature, among which the iron oxides have a strong catalytic effect on the graphitization of pitch‐based carbons. Meanwhile, the amorphous carbon region uniformly encapsulates the pitch‐derived graphitic carbon, as a result, PGC materials with different graphitization configurations can be obtained by adjusting the mixing ratio of pitch and FC.^[^ ^27^, ^28^ ^]^ The morphology of the derived carbons was investigated through scanning electron microscopy (SEM) images, as illustrated in Figure 1b–d and Figure S1 (Supporting Information). Pristine pitch pyrolytic carbon (MTP) has a lamellar‐like morphology with a bulk structure in a size of several micrometers. Introducing ferric citrate catalyst resulted in distinct morphologies for different samples where PGC11 shows the randomly distributed particles. At the same time, the PGC12 and PGC14, with higher FC content, exhibited large blocks with smooth surfaces and particle size of ≈5 µm. This distinct morphological change can be attributed to the aggregation and growth of carbon particles during the carbonization and graphitization processes. TEM images in Figure 1e–g and Figure S2 (Supporting Information) show that MTP exhibited short‐range ordered turbostratic carbon layers with an interlayer spacing of 0.348 nm based on fast Fourier transform (FFT) patterns. With the addition of FC, PGC11 shows the co‐existence of the graphite‐like region with long‐range ordered carbon layers and amorphous region, and the corresponding interlayer spacing was 0.342 and 0.350 nm, respectively. In the case of PGC12, the highly oriented carbon layers were presented with a reduced interlayer spacing of 0.337 nm and some amorphous regions with an interlayer spacing of 0.346 nm persisting within the microcrystalline structure. Further, PGC14 exhibited fully oriented carbon layers with an interlayer spacing of 0.337 nm, suggesting a nearly completely graphitized structure.

**Figure 2:**
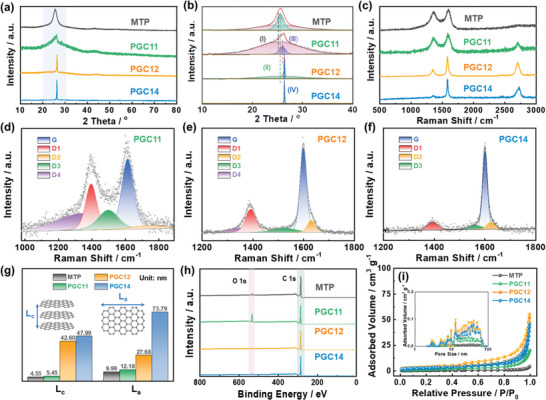
a) XRD patterns of PGCs in reference to MTP, b) corresponding deconvoluted (002) peak, c) Raman spectra, and d–f) corresponding deconvoluted profiles, g) the calculated crystal parameters, h) XPS survey spectrum, and i) N_2_ adsorption‐desorption isotherms of the derived carbons with an inset showing the pore‐size distribution histogram.

X‐ray diffraction (XRD) and Raman spectrum were utilized to further characterize the local carbon structures. **Figure** **2**a shows the XRD patterns of the derived carbons, where the diffraction peak at ≈26° corresponds to the (002) plane of carbon materials.^[^ ^29^, ^30^ ^]^ As the FC addition increases, the (002) peaks progressively get narrow. The variation in peak shape and intensities of typical (002) peaks across samples indicate their varying microstructures. To further elucidate the distinct microstructure changes, the (002) peaks for all samples were deconvoluted into secondary peaks with varying diffraction degrees. Figure 2b shows the corresponding profile comprised of peak I (≈25.3°, 0.351 nm), peak II (≈25.6°, 0.347 nm), peak III (≈26.0°, 0.342 nm), and peak IV (≈26.4°, 0.337 nm).^[^ ^31^, ^32^, ^33^, ^34^ ^]^ As seen, the (002) peak of MTP comprises peaks I and II with the largest interlayer spacing, indicating its highly disordered structure. For PGC11, the (002) peak could be deconvoluted into peak I and peak III, with peak III representing a more compressed carbon structure with a narrow interlayer spacing of 0.342 nm, indicative of increased graphitization. As the FC content further increases, the degree of graphitization enhances, resulting in the appearance of peak IV for PGC12, which is then predominant in the case of PGC14, justifying the progressive graphitization under the catalytic effect of FC.

Figure 2c presents the Raman spectrum of the carbon materials, where two typical peaks at ≈1350 and ≈1590 cm^−1^ correspond to the D peak induced by defects and the G peak associated with the crystalline graphitic phase, respectively. The spectrum was further deconvoluted into five sub‐peaks (D1, D2, D3, D4, G), as shown in Figure 2d–f and Figure S3 (Supporting Information). The D1 band is related to the vibrations of disordered carbon atoms within a defective graphitic structure or at the graphite sheets' edges (A_1g_ symmetry); D3 band is ascribed to the short‐range sp^3^ carbon vibration of amorphous carbon; D4 band is likely associated with sp^2^‐sp^3^ bonds or the disordered graphitic lattice; the G band originates from the stretching vibrations of sp^2^‐hybridized graphitic carbon atoms and D2 band near the shoulder of the G band can be assigned to the lattice vibration analogous to those of the G band but involving graphene layers on the surface of a graphitic crystal (E_2g_ symmetry).^[^ ^35^, ^36^, ^37^, ^38^ ^]^ As a representative pitch‐derived amorphous carbon, MTP exhibits a high I_D1_/I_G_ ratio of 0.93, which progressively decreases to 0.62, 0.44, and 0.24 for PGC11, PGC12, and PGC14, respectively (Table S1). This trend indicates a reduction in defect contents as the degree of graphitization increases. Additionally, the I_D3_/I_G_ value follows the same trend, which gradually reduces from 0.67 for MTP to 0.48, 0.20, and 0.16, confirming the enhanced graphitization degree with the addition of the catalyst.

Meanwhile, the carbon crystallite size, denoted as L_a_ and L_c_, was calculated based on the XRD and Raman results, as displayed in Figure 2g. L_a_ could be obtained according to the Raman result,^[^ ^33^, ^39^ ^]^ and it was found that MTP has the smallest L_a_ value of 9.99 nm, which increased to 12.18 nm for PGC11. With further increase in catalytic content (FC), the graphitization degree rises in PGC12 and PGC14, leading to significantly larger L_a_ values of 27.68 and 73.79 nm, respectively. Additionally, the crystallite size perpendicular to the basal plane, L_c_ calculated from the Scherrer formula,^[^ ^36^, ^40^, ^41^ ^]^ follows a similar trend to L_a_. After the addition of FC, the L_c_ values slightly increased from 4.55 nm for MTP to 5.45 nm for PGC11. The increase is more pronounced for PGC12 and PGC14, where L_c_ values escalate to 42.60 and 47.99 nm, respectively, indicating a significantly improved degree of graphitization and rapid growth of the crystallinity of the derived carbons owing to the strong catalytic effect of FC.

X‐ray photoelectron spectrum (XPS) was applied to examine the elemental composition and content of the derived carbons (Figure 2h). All the carbons consisted solely of C and O elements with no detectable impurity, confirming the complete removal of catalyst (Fe) in the final products. The C element content in MTP is 95.5%, which increases to 98.0% in PGC12, indicating sufficient carbonization under the catalytic effect of FC. Here, the notably high O content in PGC11 (15.8%) may result from the introduction of oxygen‐containing functional groups during the decomposition of ferric citrate (FC), and the corresponding carbonization process with poor catalytic graphitization couldn't efficiently remove the oxygen elements. Figure S4 (Supporting Information) shows the C1s spectra deconvoluted into four components at 284.6, 284.8, 285.6, and 290.0 eV, corresponding to the C═C (sp^2^‐C), C─C (sp^3^‐C), C─OH, and O─C═O, respectively.^[^ ^42^, ^43^ ^]^ The ratio of C─C to C═C in MTP is 1.23, which decreases to 0.97 for PGC11, 0.58 for PGC12, and 0.51 for PGC14, indicating an increasing degree of graphitization, corroborating the XRD and Raman results.

N_2_ (77 K) adsorption‐desorption measurement was employed to evaluate the porous structure of the pitch‐derived carbons, as shown in Figure 2i. MTP exhibits negligible N_2_ adsorption with a low specific surface area (SSA) of 1.41 m^2^ g^−1^ and a minimal pore volume of 0.0066 cm^3^ g^−1^. Following the introduction of FC, the SSA and pore volumes of PGCs exhibit slight increases to 6.07 m^2^ g^−1^ and 0.031 cm^3^ g^−1^ for PGC11, and then 13.06 m^2^ g^−1^ and 0.085 cm^3^ g^−1^ for PGC12, respectively. A further increase in FC content led to reduced SSA and pore volume in PGC14, measuring 10.67 m^2^ g^−1^ and 0.071 cm^3^ g^−1^, which might be attributed to the aggregation of carbon blocks during the excessive graphitization process. The extremely low SSA and pore volume confirm that FC did not function as a template to develop porous structures; therefore, the porosity has a negligible influence on evaluating the K‐storage performances of prepared carbons.

**Figure 3:**
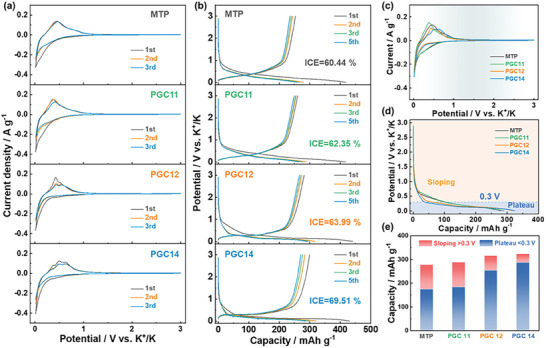
Electrochemical potassium storage behaviors and performance of PGCs. a) CV curves at a scan rate of 0.1 mV s^−1^, b) GCD curves at a current rate of 0.1 C, comparison of c) CV curves, and d) discharge curves of 2nd cycle of PGCs in reference to MTP, and e) the corresponding sloping and plateau region capacity distribution during the discharge process.

Cyclic voltammetry (CV) measurements were conducted in K‐ion half‐cell using 0.8 m KPF_6_ (EC+DEC) as the electrolyte to investigate the K‐storage behaviors of the PGC samples at a scan rate of 0.1 mV s^−1^ within the potential range of 0.01–3.0 V (vs K^+^/K), as shown in **Figure** **3**a,c. The CV curves of MTP feature a pair of reduction/oxidation peaks at ≈0.01 V (cathodic) and 0.45 V (anodic), corresponding to the intercalation/de‐intercalation processes of K‐ion between the carbon layers. Similar to MTP, PGC11 also exhibits a similar CV profile owing to a relatively low graphitization degree. However, as the graphitization degree increases in PGC12 and PG14, their CV behavior diverges from that of MTP with an obvious anodic peak at ≈0.45 V split into secondary peaks, which should be attributed to the formation of different K‐graphitic intercalation compounds (K‐GICs) within the graphitized carbon structures. Moreover, despite a slight enhancement in SSA for PGCs, the irreversible region of their CV curves gradually reduces, which can be ascribed to the diminished defects and lower SEI formation in the graphitized carbon structures during the potassium storage process.

To assess the differences in K‐storage performances of the derived PGC carbons, galvanostatic charge/discharge (GCD) tests were carried out at a current rate of 0.1 C (1 C = 279 mA g^−1^), as shown in Figure 3b. MTP, characterized by its typical soft carbon nature, delivered a reversible K‐storage capacity of 253.2 mAh g^−1^ with an initial coulombic efficiency (ICE) of 60.44% (Table S2). PGC11 displayed a similar reversible K‐storage capacity of 260.5 mAh g^−1^ and an ICE of 62.35%. The addition of FC in PGC12 led to an appreciable increase in reversible capacity to 281.9 mAh g^−1^ and an ICE of 63.99%. Further, PGC14, with the highest degree of graphitization, realized the highest reversible capacity of 299.1 mAh g^−1^, and ICE of 69.51%. The enhancement in reversible K‐storage capacity and ICE value for the PGC electrodes can be attributed to the highly developed graphitic structures, which provide abundant K‐ion intercalation sites, as well as the restricted SEI formation due to the much‐decreased defect sites within the carbon material.

The low‐potential plateau region capacity for carbon anodes plays a critical role in achieving the high capacity and output voltage in the assembled full‐cell. The capacity derived from the sloping region and plateau region of different PGC electrodes was analyzed, as presented in Figure 3d. To eliminate the interference of initial irreversible capacity, the discharge curves of the 2nd cycle were used for comparison. Notably, as the graphitic component in carbon anode increased, the potential of the K‐storage plateau region decreased, accompanied by a significant reduction in sloping region capacity. The corresponding capacities for the plateau and sloping regions were differentiated at a cut‐off potential of 0.3 V (Figure 3e). It is evident that PGC12 and PGC14, with much lower degrees of disordered and defect content, exhibited considerably low capacities of 63.1 and 37.3 mAh g^−1^ from the high‐potential sloping region (>0.3 V). However, the samples demonstrated substantial capacities of 253.5 and 287.4 mAh g^−1^ in the low‐potential region (<0.3 V). In contrast, MTP and PGC11 only achieved lower capacities of 175.8 and 183.9 mAh g^−1^ in the plateau region, respectively. These results indicate that enhancing the graphitization degree significantly improves the K‐storage capacity in the low‐potential region, which is crucial for the further development of K‐ion full cells.

**Figure 4:**
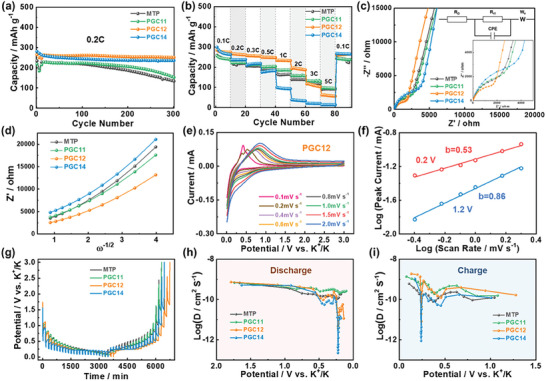
a) Cycle performance at a current rate of 0.2 C, and b) rate performance at different current rates, c) EIS spectrums, d) the relationship between Z’ and ω^−1/2^ of all the samples. e) CV curves of PGC12 at various scan rates of 0.1–2.0 mV s^−1^, and f) the correlation of current (*i*) and scan rate (*ν*) at 0.2 V in the cathodic process and 1.2 V in the anodic process. g) GITT profiles of the 2nd cycle and the corresponding K‐ion diffusion coefficients at h) discharge and i) charge processes of the derived carbons.

The cycle stability of the obtained PGC electrodes was systematically evaluated at various current rates. Under a small current rate of 0.1 C, all the derived carbons could exhibit good cycle stability and maintain well with their reversible K‐storage capacity, as displayed in Figure S5a (Supporting Information). However, an obvious cycle stability difference was observed at a slightly increased current rate of 0.2 C. MTP presented a low initial capacity of 234.5 mAh g^−1^, and its reversible capacity gradually decreased to 133.5 mAh g^−1^ after 300 cycles, resulting in a capacity retention of only 56.9% (**Figure** **4**a). Similarly, PGC11 with a slightly increased graphitization degree also exhibited capacity decay with a capacity retention of 65.2% after 300 cycles. This decay likely originates from the continuous formation of the SEI layer at the defects or edges on the amorphous carbon structure, resulting in the failure of active sites and a gradual reduction in reversible capacity during the charge/discharge processes. In contrast, PGC12, with a higher degree of graphitization and a partial amorphous carbon structure, maintained the highest capacity of 251.0 mAh g^−1^ with a significantly improved capacity retention of 94.8% after 300 cycles. In comparison, the slightly lower capacity retention of 83.9% for PGC14 can be ascribed to its excessive graphitization that generates certain structural damage during the K‐storage process. Furthermore, PGC12 also shows its prominent advantage at a current rate of 0.5 C, maintaining a high capacity of 216.6 mAh g^−1^ after 200 cycles, as shown in Figure S5b (Supporting Information). The inferior performance of PGC14 at 0.5 C might be attributed to the substantial volume change and sluggish kinetic in the highly graphitized carbon structure. The comparative assessment confirms the advantage of the unique carbon configuration that combines a highly graphitized structure with a partially amorphous region in offering the significantly increased K‐storage capacity and enhanced reversibility of PGC12. The highly graphitized part provides abundant interlayer intercalation sites for K ion storage, thereby extending the plateau region capacity, while the amorphous region acts as a volume buffer space and contact intermediaries between the graphitized structure and the charge carrier, which could significantly reduce the distance of K‐ion migration and improve the reaction kinetics, optimizing the overall battery performance.

Excessive graphitization negatively impacts the ion diffusion/transport, resulting in severe capacity degradation at high current rates, as shown in Figure 4b. Despite the highest reversible capacity at the current rate of 0.1 C, PGC14 demonstrated the worst rate performance with the capacity sharply declining to 98.5 mAh g^−1^ at 1 C, and almost no capacity retained when the current rate increases to 5 C. This degradation is primarily due to the sluggish K‐ion transport kinetic resulting from a mismatch between the large ionic radius of K‐ion and the small interlayer spacing within the compact carbon structure of PGC14. Interestingly, due to the existence of an amorphous carbon phase, the PGC12 maintained relatively high reversible K‐storage capacities of 222.2 and 179.5 mAh g^−1^ at 1 and 2 C, respectively. However, further increasing the current rate, PGC12 shows a noticeably deteriorated capacity with only 101.0 and 55.1 mAh g^−1^ retained at large current rates of 3 and 5 C. Figure S6 (Supporting Information) compares the variation in charge/discharge curves of the PGC12 electrode as the current increases. At a current rate of 3 C, the charge/discharge curves predominately exhibit a sloping region, indicative of the adsorption process of K‐ion at the edge, defect, etc. Thus, the combination of lower defect content and a relatively high degree of graphitization may account for the observed performance deterioration of PGC12 at increased current rates.

Electrochemical impedance spectroscopy (EIS) was utilized to analyze the electrochemical kinetics of the prepared carbons, as shown in Figure 4c. The EIS‐based Nyquist plot features semicircles in the high‐frequency region and a nearly vertical line in the low‐frequency region, representing the charge transfer resistance (*R*_ct_) and ion diffusion resistance of the electrode, respectively. PGC12 delivers a much lower *R*_ct_ value of 1833 than 2344 Ω for MTP, reflecting the improved reaction kinetics within the carbon structure. Figure 4d shows the relationship between Z’ and ω^−1/2^,^[^ ^44^, ^45^ ^]^ aiding in the analysis of ion‐diffusion dynamics. The calculated potassium diffusion coefficient of PGC12 was 4.6 × 10^−13^ cm^2^ s^−1^, which significantly surpasses those of MTP (1.0 × 10^−13^ cm^2^ s^−1^), PGC11 (1.9 × 10^−13^ cm^2^ s^−1^), and PGC14 (0.6 × 10^−13^ cm^2^ s^−1^), confirming the rapid ion diffusion process in PGC12 electrode.

CV measurements at various scan rates from 0.1 to 2.0 mV s^−1^ were measured to investigate the electrochemical K‐storage mechanism and kinetics of the derived PGC carbons (Figure S7, Supporting Information). The relationship between the current (*i*, mA) and the scan rate (*ν*, mV s^−1^) is expressed by the equation *i* = *aν^b^*. The *b*‐value of 1.0 represents a capacitive‐controlled behavior, while 0.5 indicates a diffusion‐controlled behavior.^[^ ^46^, ^47^, ^48^, ^49^ ^]^ As depicted in Figure 4e–f, PGC12 exhibited a b‐value of 0.53 at 0.2 V during the cathodic process, indicating the diffusion‐dominated process at the low potential range, corresponding to the K‐intercalation in its highly graphitized structure. Conversely, the *b*‐value of 0.86 at 1.2 V during the anodic process indicates a predominately capacitive‐controlled mechanism in the high potential region, which originated from the K‐adsorption at the defect or edge sites.

In order to further investigate the K‐ion storage kinetics, the K‐ion diffusion coefficient (D_K+_) in various carbon structures was analyzed using the galvanostatic intermittent titration technique (GITT) test. Figure 4g displays the GITT profiles with a pulse current rate of 0.1 C for the derived carbons. The corresponding D_K+_ was calculated by using the following equation:^[^ ^50^, ^51^, ^52^ ^]^

(1) $$\;D_{K}=\frac{4}{\pi \tau }\;\left(\frac{m_{B}V_{M}}{M_{B}S}\right){\left(\frac{\Delta E_{S}}{\Delta E_{\tau }}\right)}^{2}$$

where τ is the pulse time (s), m_B_ is the mass of active material, M_B_ is the molar mass of the active material, V_M_ is the molar volume of the active materials, S is the electrochemically active surface area of the electrode, ΔE_S_ is the steady‐state voltage change, and ΔE_τ_ is the voltage change during current pulse application (Figure S8, Supporting Information). Figure 4h,i shows the variation of D_K+_ with potential during discharge and charge stages in the 2nd cycle. Here, MTP and PGC11 with their relatively amorphous carbon structure, realized the higher D_K+_ value with some small peaks appeared during the discharge/charge processes, which should be attributed to the storage of K‐ion between the amorphous carbon layers by forming the primitive K‐GICs. In contrast, PGC12 showed dramatically different D_K+_ curves, where the abrupt appearance of the D_K+_ peaks at the potential ≈0.25 and 0.50 V can be attributed to the low migration kinetic of K‐ion within the graphitized carbon structures and a significant increase in the formation of K‐GICs. Additionally, PGC14, with the highly graphitized structure, delivered more pronounced D_K+_ peaks and reduced D_K+_ value across the entire potential window, suggesting the decreased diffusion rate in the highly graphitized carbon structures.

**Figure 5:**
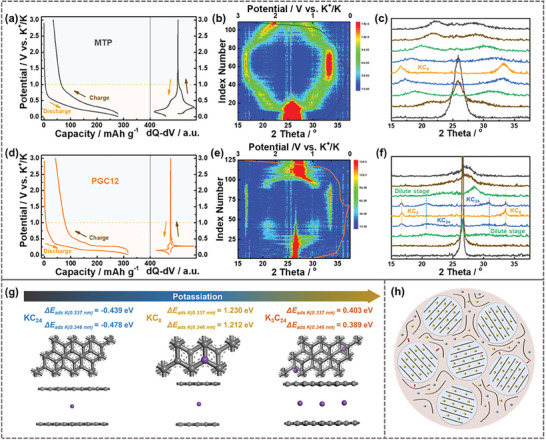
GCD and the corresponding dQ‐dV curves along with in‐situ XRD patterns in the first cycles of a–c) MTP and d–f) PGC12. g) Theoretical simulations for the adsorption energy of potassium adsorption on the interlayer sites in various configurations, specifically KC_24_, KC_8_, and K_3_C_24_. Gray and purple balls represent carbon and potassium atoms, respectively. h) Schematic illustration depicting the optimal carbon structure for high‐performance potassium storage.

To further monitor the structure transformation and clarify the K‐ion storage mechanism during the charge/discharge process, dQ–dV analysis and in‐situ XRD techniques were performed for the first cycle of the derived PGC12 electrodes with the inference of MTP, as shown in **Figure** **5**a**–**f and Figures S9–S11 (Supporting Information). The dQ–dV curves indicate that the main capacity contribution of the derived carbon stems from the potential region below 1.0 V, which corresponds to the K‐storage in well‐developed graphitic structures. Unlike MTP and PGC11, which display only a pair of broadened peaks, PGC12 and PGC14 exhibit sharper peaks with the existence of some smaller peaks attributed to the formation of K‐graphitic intercalation compounds (K‐GICs). In‐situ XRD patterns reveal the evaluation of the microcrystalline structure during the potassiation/de‐potassiation process. As seen, the (002) peak intensity can be well maintained in the initial discharge stage until to 1.0 V, corresponding to the adsorption‐storage process along the high‐potential region. With the proceeding of the discharge stage, the intensity of (002) peaks decreases progressively for both MTP and PGC12, with paired peaks emerging on both sides, corresponding to the formation of the K‐GICs. Here, MTP undergoes a continuous structural transformation from the (002) peak into the specific peaks of KC_8_ (16.55°/33.42°), corresponding to the pair of broadening peaks at 0.15/0.30 V in the dQ–dV curve. In contrast, PGC12, with the significantly higher graphitization degree exhibits a staged phase transition: KC_x_ (early stages at 1.0–0.20 V, KC_144‐168_, KC_48_ and KC_36_) to KC_24_ (0.20–0.15 V, 20.45°/30.81°) and then to KC_8_ (0.15–0.01 V, 16.56°/33.56°), as displayed in Figure 5e,f. During the charge process, a reversed structural transition occurs, indicating the good reversibility of the structure changes. The formation of KC_24_ and KC_8_ correspond to the peaks at ≈0.21/0.40 V and 0.14/0.28 V in the dQ–dV curve, respectively. The difference in the phase transition process indicates the distinct storage mechanisms of K‐ion in graphitic carbon versus amorphous carbon, where the highly graphitized carbon exhibits a staged phase transition during the K‐storage process, while the amorphous carbon shows a continuous structural transition for K‐ion storage.

Density functional theory (DFT) calculations were conducted to further investigate the potassium storage properties in different carbon configurations, as illustrated in Figure 5g. For the configurations of KC_24_ and KC_8_, the Δ*E*_ads_ of potassium in the graphitic carbon model (interlayer spacing of 0.337 nm) are −0.439 and 1.230 eV, respectively. These values are higher than −0.478 and 1.212 eV for the amorphous carbon model (interlayer spacing of 0.346 nm). This change in Δ*E*_ads_ further supports the notion that the increased interlayer spacing will facilitate the storage of potassium in different K‐GICs. Then, the C_24_ model was further applied to store three potassium atoms to explore the K‐GICs configuration of K_3_C_24_ (3KC_8_). Interestingly, the Δ*E*_ads_ of each potassium atom (KC_8_) in K_3_C_24_ are 0.403 eV for graphitic carbon (0.337 nm) and 0.389 eV for amorphous carbon (0.346 nm), respectively. The considerably lower Δ*E*_ads_ for K_3_C_24_ compared to the configuration of single KC_8_ (1.230 eV for 0.337 nm and 1.212 eV for 0.346 nm) implies the preference for K‐storage in the graphitic carbons with larger crystallite size, facilitating the potassium storage in carbon structures. Thus, it is evident that both enlarged interlayer spacing and increased crystallite size in carbon materials are beneficial for storing potassium within the carbon layers.

However, it is critical to recognize that interlayer spacing, graphitization degree, and crystallite size are not independent variables in carbon materials. The amorphous carbon, characterized by larger interlayer spacing, typically exhibits a smaller crystallite size. Conversely, highly graphitized carbon, with its well‐developed crystallite structures, tends to have a significantly reduced interlayer spacing. Here, an optimal structural model of carbon anodes for high‐performance PIBs was proposed, as illustrated in Figure 5h. The model integrates a highly graphitized main‐body structure that offers abundant intercalation sites for K‐ion storage, paired with an appropriate amorphous region, which acts as the buffer to accommodate volumetric expansion of the graphitic structure but also enhances ion diffusion kinetics. By leveraging the synergistic effect of the graphitized and amorphous regions, the potassium‐storage capabilities of carbon anodes can be substantially improved, paving the way for more efficient and durable battery technologies.

**Figure 6:**
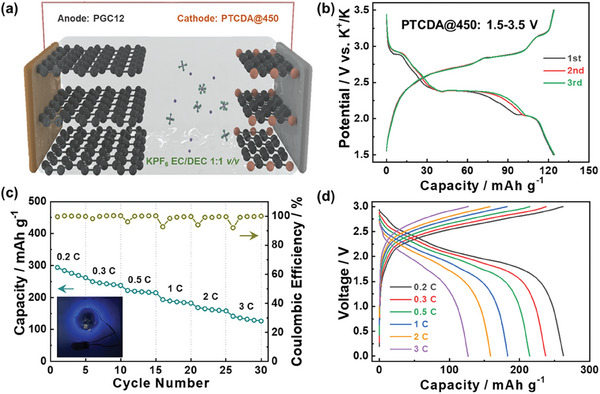
a) Schematic illustration of the potassium‐ion full‐cell with PGC12 as anode and PTCDA@450 as cathode, b) GCD curves of PTCDA@450, c) the rate capability, and d) the corresponding GCD curves at different current rates for the assembled full‐cell.

To evaluate the practical applicability of the developed PGC anode material, a potassium‐ion full‐cell was assembled with PGC12 as an anode and PTCDA@450 as a cathode,^[^ ^53^, ^54^ ^]^ as illustrated in **Figure** **6**a. Both the PGC12 anode and PTCDA@450 cathode were pre‐cycled in half‐cell setups before being configurated into the full‐cell, and the charge/discharge curves of PTCDA@450 within the voltage window of 1.5–3.5 V are shown in Figure 6b. The assembled PTCDA@450//PGC12 full‐cell demonstrated a reversible capacity of 262.1 mAh g^−1^ based on the mass of the carbon anode at a current rate of 0.2 C. Moreover, the energy density was estimated to be 148.2 Wh kg^−1^ based on the total mass of the anode and cathode materials. The practical functionality of the cell is highlighted by its ability to power an LED, shown in the inset of Figure 6c. Additionally, the cell maintained a high capacity of 126.7 mAh g^−1^ when the current rate increased to 3 C (Figure 6d). The robust charge/discharge behavior at elevated current rates further verified the promising prospect of PGC12 as an anode material for PIBs.

## 3 Conclusion

Herein, a series of carbon materials with varying graphitic‐amorphous architectures were successfully synthesized using pitch as a carbon source and ferric citrate as a graphitization catalyst, to elucidate the relationship between structural configuration and the electrochemical K‐storage properties in carbon anodes. Although highly graphitized carbon offers numerous active sites for K‐ion intercalation, contributing to enhanced capacity in the low‐potential plateau region, excessive graphitization compromised the electrochemical performance at high rates. In contrast, the presence of the amorphous carbon phase provides a buffer space that accommodates volume expansion of the graphitized region as well as additional adsorption sites, which offer the capacity in sloping regions and meanwhile improve the rate capability. As a consequence, the partial graphitic carbon (PGC) configuration was proposed with the integration of the graphitic region and amorphous phase to achieve improved electrochemical K‐storage performance. The optimum PGC12 electrode demonstrated a reversible capacity of 281.9 mAh g^−1^ as well as excellent cycle and rate capability with a retained capacity of 179.5 mAh g^−1^ at 2 C. Furthermore, the assembled K‐ion full‐cell could deliver a reversible capacity of 262.1 mAh g^−1^ at 0.2 C and an energy density of 148.2 Wh kg^−1^. The insight into the K‐ion storage mechanism in graphitic and amorphous phases will deepen the understanding of the structure‐property correlation of carbon anode, which may greatly accelerate the commercial development of high‐performance carbon materials for potassium‐ion batteries.

## Conflict of Interest

The authors declare no conflict of interest.

## Supporting information



Supporting Information

## Data Availability

The data that support the findings of this study are available from the corresponding author upon reasonable request.
