# Supplementary material for: Rigid‐Flexible Coupling Realized by Synergistic Engineering of the Graphitic‐Amorphous Architecture for Durable and Fast Potassium Storage
Source: Adv Sci (Weinh). 2024 Nov 22;12(3):2410966. doi: 10.1002/advs.202410966 (PMC11744585; doi:10.1002/advs.202410966)
Supplement: Supplementary file 1 — Supporting Information [file ADVS-12-2410966-s001.docx]

Supporting Information

Rigid-Flexible Coupling Realized by Synergistic Engineering of the Graphitic-Amorphous Architecture for Durable and Fast Potassium Storage

Mingchi Jiang, Ning Sun*, Bin Cao, Xuyang Jian, Razium Ali Soomro, Bin Xu*

**Experimental section**

***Materials Synthesis:*** The partial graphitic carbons (PGC) were synthesized using coal tar pitch as carbon precursor and ferric citrate (FC) as graphitization catalyst. The coal tar pitch powder and FC were uniformly mixed in a certain mass ratio, and the obtained mixture was then carbonized at 1400 °C for 2 h under an Ar atmosphere with a heating rate of 5 °C min^-1^. The derived carbonized product was washed with 3 M HCl (dilute hydrochloric acid) and rinsed with deionized water until neutral to obtain the final partial graphitic carbon (PGC). According to the mass ratio of pitch and ferric citrate, the resultant carbons are labeled PGC11, PGC12, and PGC14, respectively. For comparison, pristine pitch-derived carbon (MTP) was obtained by directly pyrolyzing coal tar pitch powder at 1400 °C for 2 h under Ar atmosphere without additional materials.

***Materials Characterizations****:* The microcrystalline structural characteristics of the derived partial graphitic carbons were analyzed by powder X-ray diffraction (XRD, Rigaku Ultima IV) with Cu-K𝛼 radiation (λ=1.5405 Å) and Raman spectroscopy (Renishaw inVia Reflex spectrometer). Scanning electron microscope (SEM, HITACHI S4800) and field emission transmission electron microscope (TEM, FEI Tecnai G2 F30) were employed to observe the morphology and crystalline structure of the derived carbon materials. The nitrogen adsorption-desorption isotherms were measured using a Micromeritics ASAP2460 analyzer. The surface chemical composition and elemental states were investigated through X-ray photoelectron spectroscopy (XPS, Thermo-VG Scientific ESCALAB 250) analysis.

***Electrochemical Measurements****:* CR2025 coin cells were used to investigate the electrochemical performance of the obtained carbons. The working electrodes were prepared by spreading the homogeneous slurry of active materials, super-P and carboxymethyl cellulose (CMC), with a mass ratio 8:1:1 onto the copper foil current collector. After being dried at 120 ^o^C for 12 h under vacuum and punched into small disks with a diameter of 8 mm, the electrode was obtained, and the mass loading of active materials was kept at about 1.0 mg cm^-2^. The half-cell was assembled at an argon-filled glove box with potassium foil as the counter electrode, glass fiber as a separator, and 0.8 M KPF_6_ in ethylene carbonate (EC) and diethyl carbonate (DEC) with a volume ratio of 1:1 as electrolyte. The galvanostatic charge/discharge and galvanostatic intermittent titration technique (GITT) tests were conducted at a current rate of 0.1 C (1 C=279 mA g^-1^) on the multi-channel battery test system (Neware CT-4008T) within the potential window of 0.01-3.0 V (vs. K^+^/K) at room temperature. Cyclic voltammetry (CV) and electrochemical impedance spectroscopy (EIS) over the frequency range from 0.01 to 10^5^ Hz were tested on the BioLogic VSP electrochemical workstation. The calculation of the ion diffusion coefficient based on EIS uses the following equation:

$\text{D=}\frac{\text{R}^{\text{2}}\text{T}^{\text{2}}}{\text{2}\text{A}^{\text{2}}\text{n}^{\text{4}}\text{F}^{\text{4}}\text{C}^{\text{2}}\text{σ}^{\text{2}}}$ (S1)

Where A is the surface area of the electrode, n is the number of electrons transferred in the reaction, F is the Faraday constant, C is the concentration of potassium ions in the electrodes, and σ is the slope of Z’~ω^-1/2^.

***Potassium-ion Full-cell:*** The potassium-ion full cell was assembled using PTCDA@450 as cathode materials, which were prepared by heating treatment of 3,4,9,10-tetracarboxylic dianhydride at 450 °C for 3h under Ar atmosphere. The cathode was obtained by casting the slurry consisting of PTCDA@450, super-P, and CMC with a mass ratio 8:1:1 onto the aluminum foil and vacuum-drying at 120 ^o^C for 12 h. The PTCDA@450 electrode was also cut into a circular plate with a diameter of 8 mm, and the mass ratio of the cathode to anode is about 3.5:1. Before assembling the full cell, the PTCDA@450 cathode and PGC12 anode were pre-cycled in a K-ion half-cell for 3 cycles to eliminate the initial irreversible process. Then, the full cells were assembled in an argon-filled glove box using 0.8 M KPF_6_ in EC/DEC (1:1 v/v) as electrolyte. The galvanostatic charge/discharge test was conducted on the multi-channel battery test system (Neware CT-4008T) at room temperature.

***Theoretical simulation***: The theoretical simulations were performed via density functional theory (DFT) and the electronic structures were investigated in Dmol^3^ module of Materials Studio 2020. The PWC exchange-correlation function was set to local gradient close to LDA and the dual digital basis DND 4.4 including the d polarization function were adopted to calculate the adsorption energies and differential charge densities. The electronic energy was considered to be self-consistent when the energy change was less than 10^-5^ eV. The geometry optimization was considered to be convergent when the energy change was less than 0.1 eV Å^-1^. Horizontal graphene with a length and width of 2.5 × 4.9 Å and 16.7 × 4.9 Å were established for C_8_ and C_24_ configurations, respectively. The adsorption energy of K (*ΔE_ads_*) could be obtained from the following equation:

*ΔE_ads_ = E_total_ – E_substrate_ – E_K_* (S2)

Where *E_total_* is the total energy of the materials after the adsorption of K atoms; *E_substrate_* and *E_K_* are the energy of the bare substrate and K atom, respectively.


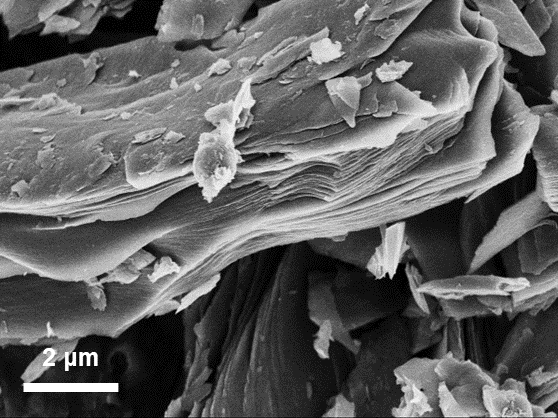


**Figure S1** SEM image of MTP.


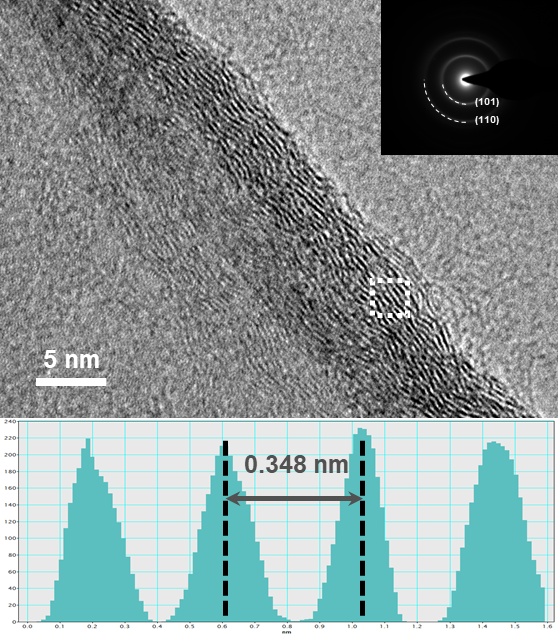


**Figure S2** HRTEM, SAED images, and the corresponding interlayer spacing measurement of MTP.


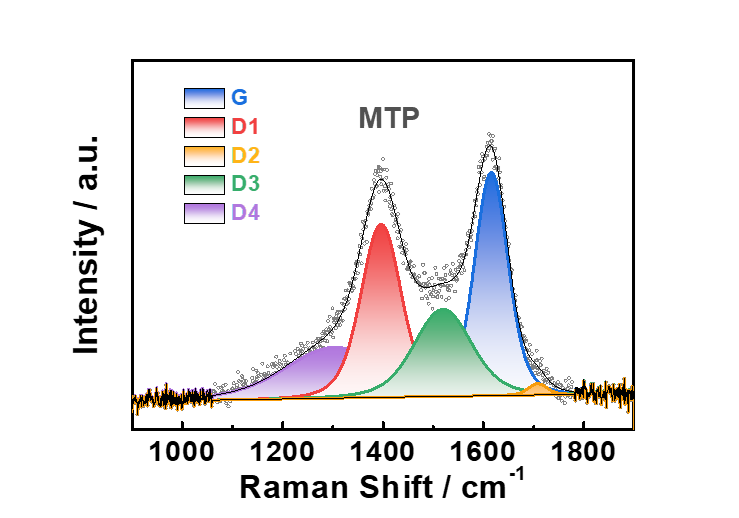


**Figure S3** The deconvoluted Raman spectra of MTP.


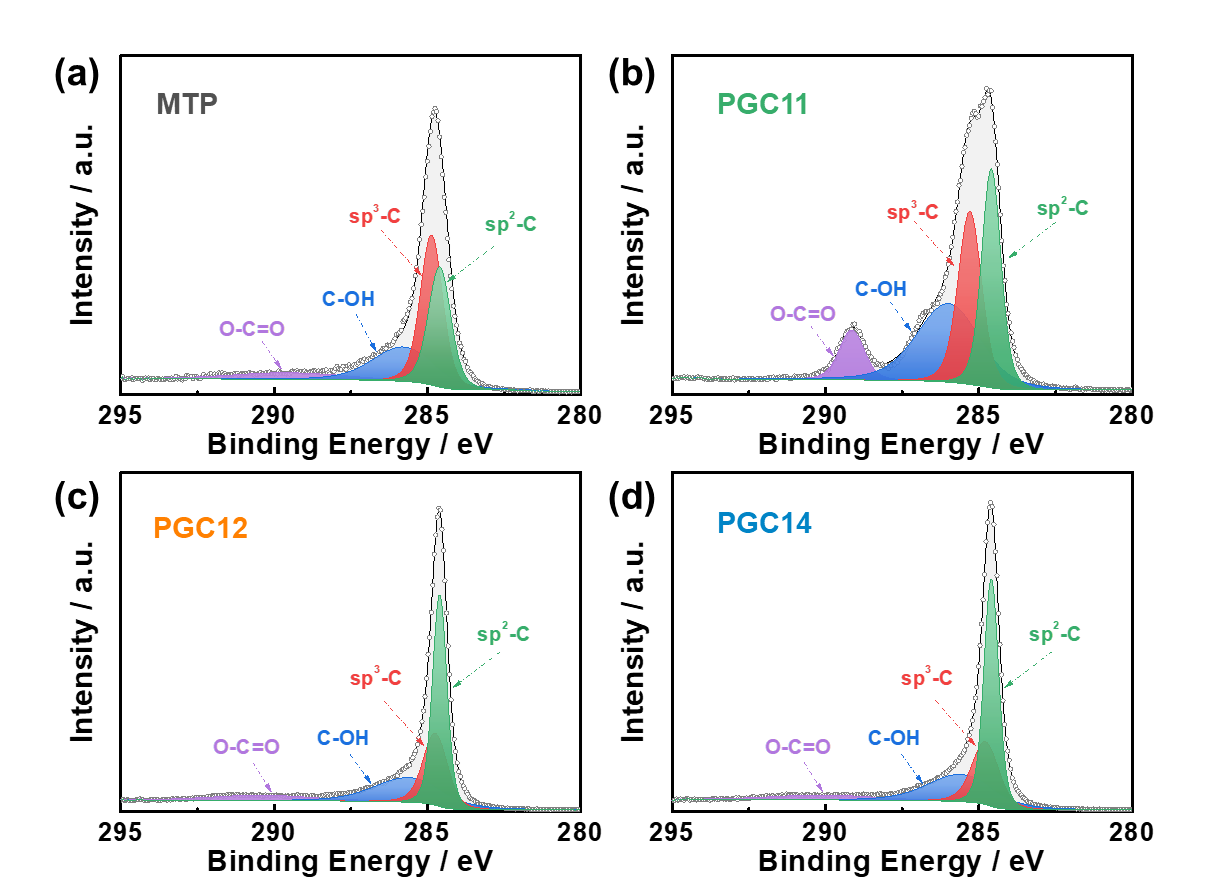


**Figure S4** The deconvoluted XPS curves


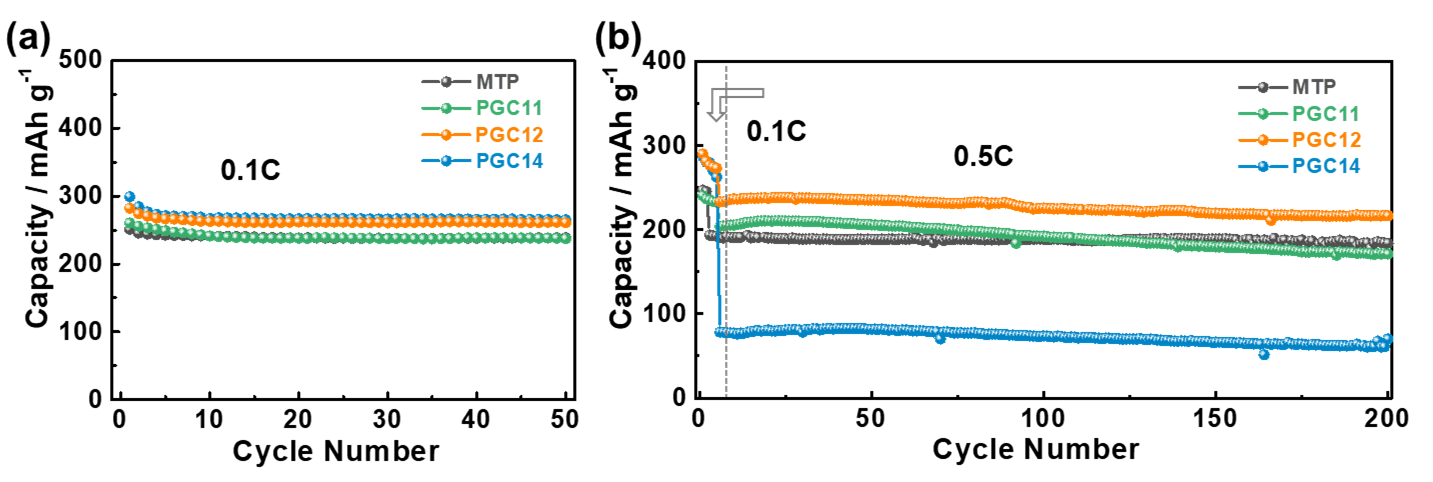


**Figure S5** The cycle stability of the pitch-derived carbons at a current rate of a) 0.1 C and b) 0.5 C.


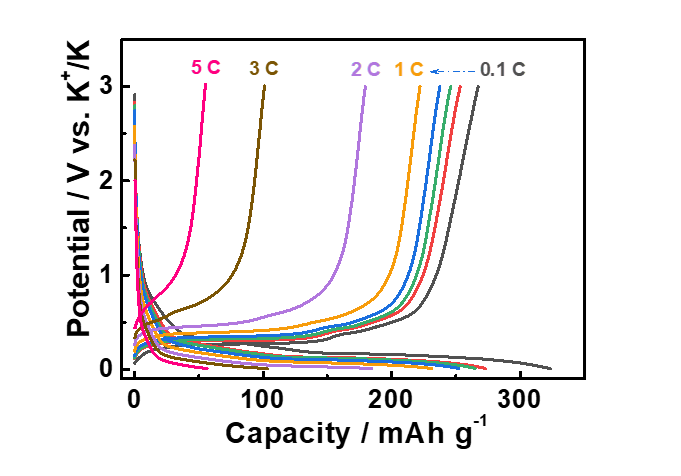


**Figure S6** The GCD curves at different current rates of PGC12.


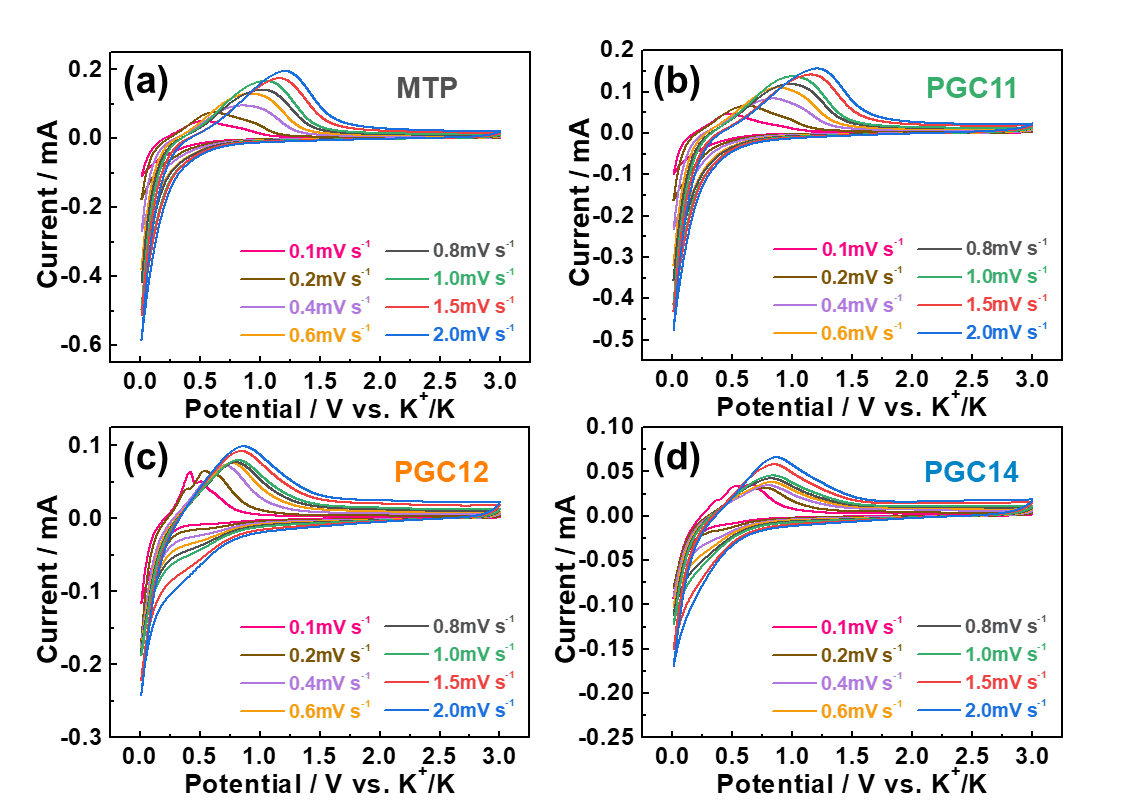


**Figure S7** CV curves at various scan rates of 0.1-2.0 mV s^-1^ of the derived carbons.


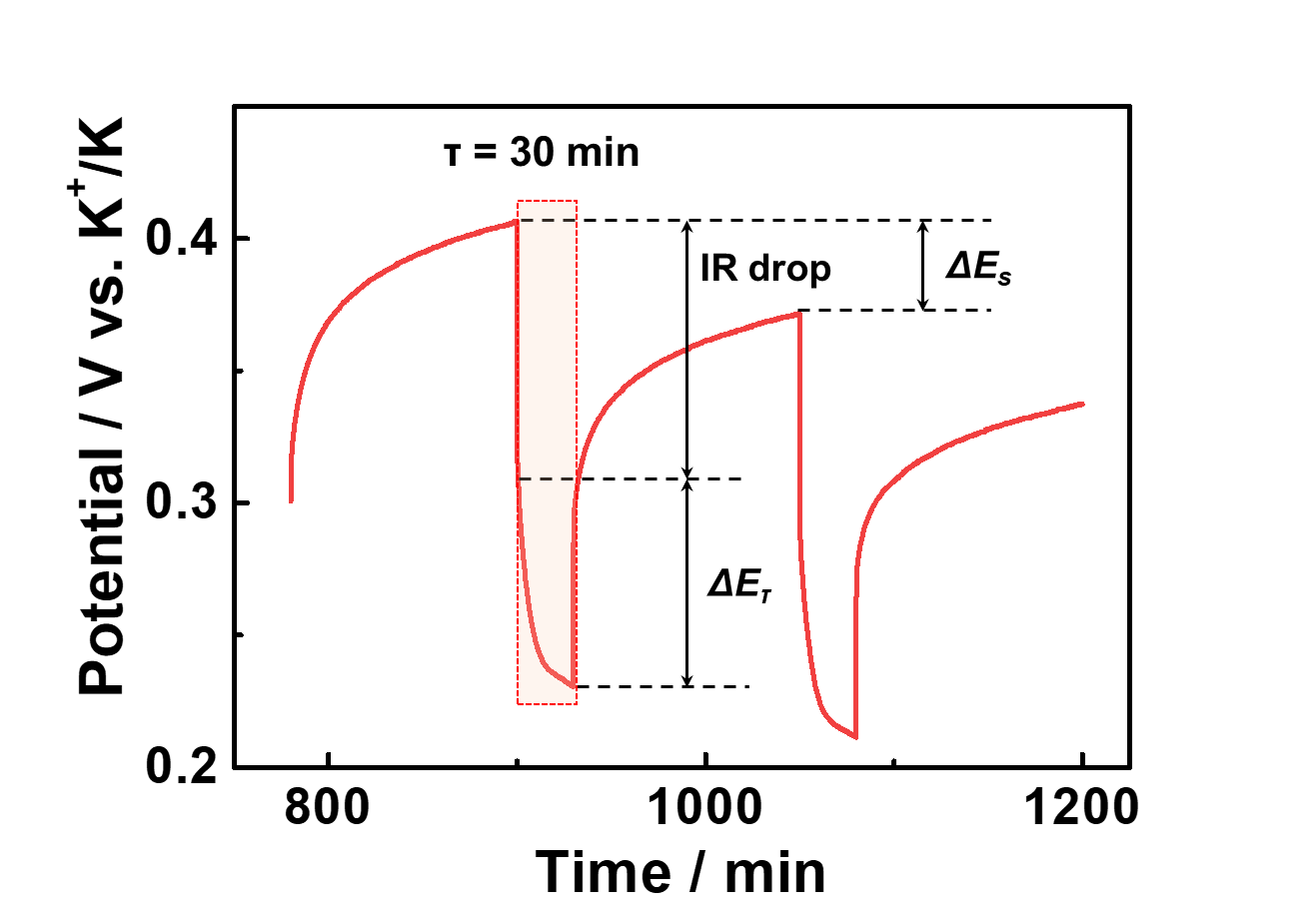


**Figure S8** Schematic for the calculation of diffusion coefficient (D_K+_) using the GITT technique.


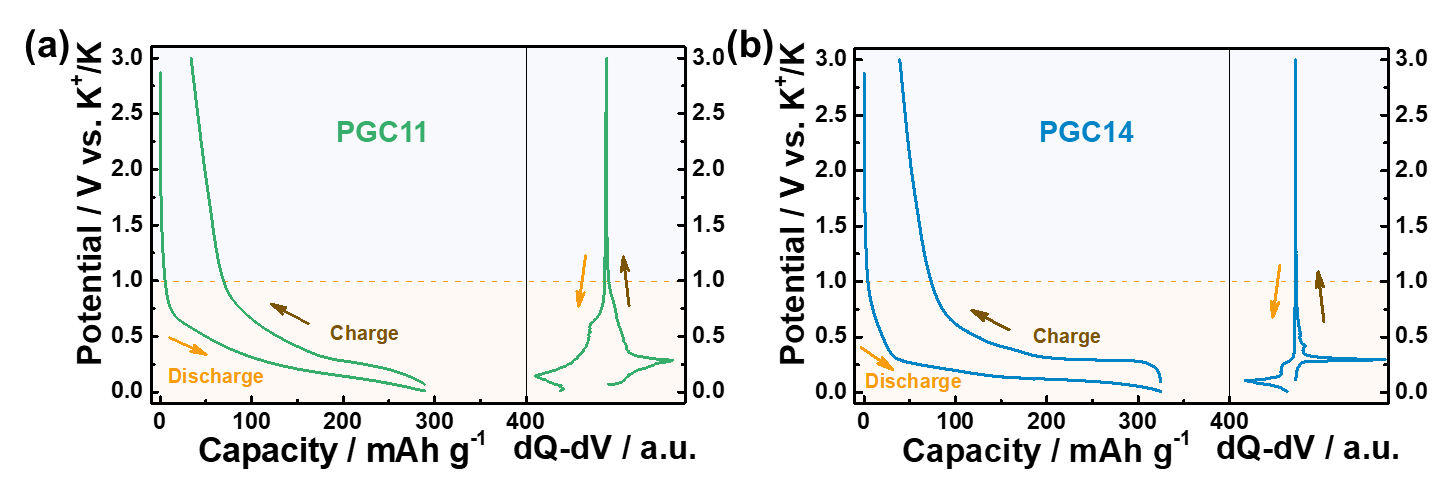


**Figure S9** GCD and corresponding dQ-dV curves in the first cycles of a) PGC11 and b) PGC14.

**Figure S10** The *in-situ* XRD test for the initial two cycles of MTP anode.

**Figure S11** The *in-situ* XRD test for the initial two cycles of PGC12 anode.

**Table S1** Structural parameters for all the graphitic carbons

|  | **d_002_ / nm** | | **L_a_ / nm** | **L_c_ / nm** | **I_D1_/I_G_** | **I_D3_/I_G_** | **SSA / m^2^ g^-1^** |
| --- | --- | --- | --- | --- | --- | --- | --- |
|  | **Region-I** | **Region-II** |  |  |  |  |  |
| **MTP** | 0.351 | 0.347 | 9.99 | 4.55 | 0.93 | 0.67 | 1.41 |
| **PGC11** | 0.351 | 0.342 | 12.18 | 5.45 | 0.62 | 0.48 | 6.07 |
| **PGC12** | 0.347 | 0.337 | 27.68 | 42.60 | 0.44 | 0.20 | 13.06 |
| **PGC14** | - | 0.337 | 73.79 | 47.99 | 0.24 | 0.16 | 10.67 |

L_a_ value was obtained according to the Raman result based on the equation: $\text{L}_{\text{a}}\text{=2.4×}\text{10}^{\text{-10}}\text{×}\text{λ}^{\text{4}}\text{×}\left( \frac{\text{I}_{\text{G}}}{\text{I}_{\text{D}}} \right)$ , where λ is the wavelength of the used Raman laser (514 nm); I_G_/I_D_ is the ratio between the integrated intensities of D and G bands in the Raman spectrums. L_c_ was obtained by the Scherrer formula: $\text{L}_{\text{c}}\text{=}\frac{\text{k}\text{λ}}{\text{β}\cos\text{θ}}$ , where k is the Scherrer constant (0.90 for L_c_); λ is the wavelength of the X-ray (0.15406 nm); β is the full width at half maxima (FWHM) of the diffraction peak; θ is the diffraction degree of the (002) peak.

**Table S2** Comparison of the electrochemical performances of the graphitic carbons

|  | **Reversible capacity / mAh g^-1^** | **ICE / %** | **Capacity / mAh g^-1^** | | **Capacity**  **at 1 C / mAh g^-1^** |
| --- | --- | --- | --- | --- | --- |
|  |  |  | **300 cycles**  **at 0.2 C** | **200 cycles**  **at 0.5 C** |  |
| **MTP** | 253.2 | 60.44 | 133.5 | 183.8 | 163.4 |
| **PGC11** | 260.5 | 62.35 | 153.1 | 171.0 | 185.6 |
| **PGC12** | 281.9 | 63.99 | 251.0 | 216.6 | 222.2 |
| **PGC14** | 299.1 | 69.51 | 235.9 | 60.2 | 98.5 |
